# Surface wave reflection from a metasurface termination

**DOI:** 10.1038/s41598-021-90429-7

**Published:** 2021-06-08

**Authors:** S. J. Berry, A. P. Hibbins, J. R. Sambles

**Affiliations:** 1 QinetiQ Ltd., Cody Technology Park, Farnborough, GU14 0LX UK; 2 Electromagnetic and Acoustic Materials Group, Department of Physics and Astronomy, University of Exeter, Exeter, EX4 4QL UK

**Keywords:** Applied physics, Techniques and instrumentation

## Abstract

The reflection coefficient of a microwave surface wave incident at the termination of a metasurface is explored. Two different surface types are examined. One is a square array of square metallic patches on a dielectric-coated metallic ground plane, the other a Sievenpiper ‘mushroom’ array. In the latter the surface wave fields are more confined within the structure. Comparison of the measured surface-wave reflection spectra is made with that obtained from analytic theory and numerical modelling. The reflection coefficient is shown to be dependent on both the momentum mismatch between the surface wave and the freely propagating modes as well as the different field distributions of the two modes.

## Introduction

The determination of the reflection coefficient of an electromagnetic surface wave incident upon a discontinuity in a surface is important in characterising the performance of surface wave structures and also for the control of radar cross sections. Studies have analytically considered the reflection coefficient of surface waves for many different types of reflecting interface^1–10^. However each solution employs different assumptions to solve the analytical equations and these assumptions introduce limitations on their applicability to describe the systems investigated within this study. Most importantly, the geometry of the metasurface structure is never fully considered. Previous works have considered the guiding surface to be a plasmonic metal^5,6,8,9^ or represented it by an impedance boundary condition^1–3,7^. Studies on the reflection coefficient of a surface wave incident upon a termination to free space was considered by Chu et al. in the 1960’s^1–3^, where the reflection and scattering to free space of a surface wave was calculated and measured experimentally. An impedance approximation was applied to the guiding interface in order to provide the necessary boundary condition for supporting a surface wave, whereas in the experiment itself a dielectric-coated metallic ground plane was used as the surface wave guiding interface^1^. Analytically the reflection coefficient of surface waves has been considered for step changes in the guiding interface^8^, changing the guiding interface impedance^7^, changing the dielectric half space^4–6^, gaps in the guiding interface^9^ and introducing a finite height overlayer discontinuity^10^. Experimentally, however, there has been little published work in the open literature, and while the studies above utilise an impedance boundary condition, a full-field analysis of the propagation of surface waves on structured metasurfaces appears to be lacking.

The purpose of this present study is to experimentally explore the reflection of microwave surface waves at the termination of two different metasurface structures, and to then compare the results with those obtained by finite element method (FEM) modelling as well as analytic theory. Furthermore, the scattered electromagnetic field at the termination is also experimentally characterised and compared with FEM modelling. It is shown that the primary scattered radiation is in the form of a forward-scattered lobe of power, scattered at small angles with respect to the plane of the metasurface.

**Figure 1:**
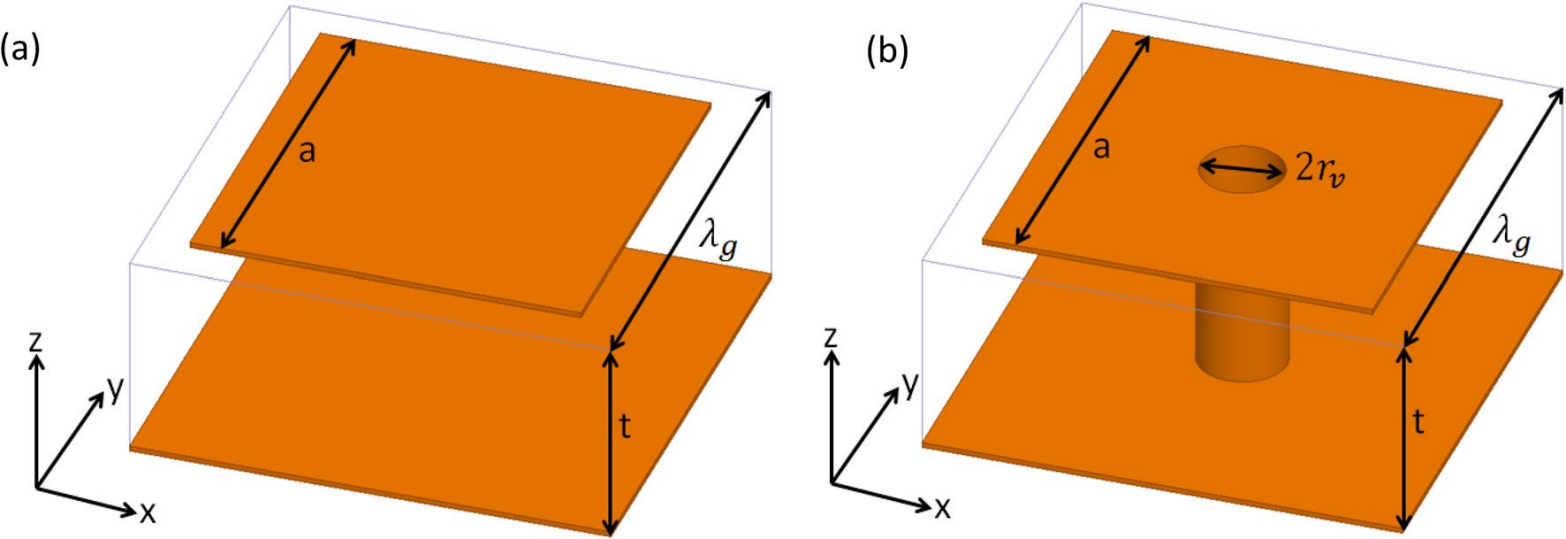
Schematic of a unit cell of the metasurface structures used in this study where the orange represents copper and the box outline indicates the dielectric; (**a**) ‘patch array’: a square array of square metallic patches on a dielectric coated metallic ground plane and (**b**) ‘mushroom’ array: the patch is connected to the ground plane with a hollow metal via.

**Figure 2:**
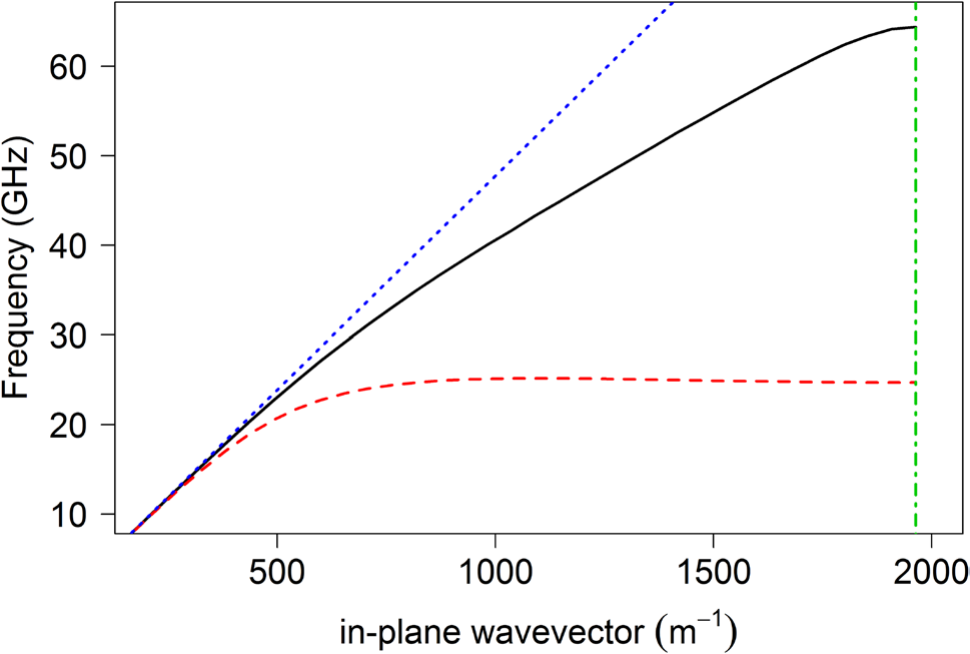
FEM predictions of the dispersion of the TM surface mode for the square array of square metallic patches on a dielectric-coated metallic ground plane (black line) and a ‘mushroom’ array (red dashed line). The dispersion of a grazing photon, the light line, is shown (blue dotted line), as is the Brillouin Zone boundary (green vertical dash-dot line) arising from the periodicity of the lattice.

Localised electromagnetic surface waves are supported on many different metasurfaces at microwave frequencies^11–15^, with the dispersion of these surface waves being defined by the geometry and materials forming the individual elements of the array (“meta-atoms”). One of the first studies of surface waves on metasurfaces was undertaken by Sievenpiper who, in his doctoral thesis, explored the dispersion of transverse-magnetic (TM)-polarised surface waves supported by so called ‘mushroom’ or ‘thumbtack’ arrays, as well as a simpler structure without the via connecting the ground plane to the patch^15,16^. In this present study these types of metasurface are used to study the reflection of surface waves at a free space termination. Unit cells for each of the two structures explored are shown in Fig. 1 and the surface wave dispersions, predicted using the eigenmode solver in a finite element method (FEM) numerical model^17^, for both metasurfaces are shown in Fig. 2. The pitch of the square array is ${\lambda }_{\mathrm{g}}=1.6$ mm, the side length of the patches is *a* = 1.3 mm and the dielectric thickness *t* = 787 $\mu m$ in both cases while the via radius ${r}_{\mathrm{v}}=150$ $\mu m$. Arrays of area 60 cm by 40 cm were fabricated using standard print-and-etch circuit-board techniques, and experimentally characterised and explored over the frequency range 10 to 30 GHz.

**Figure 3:**
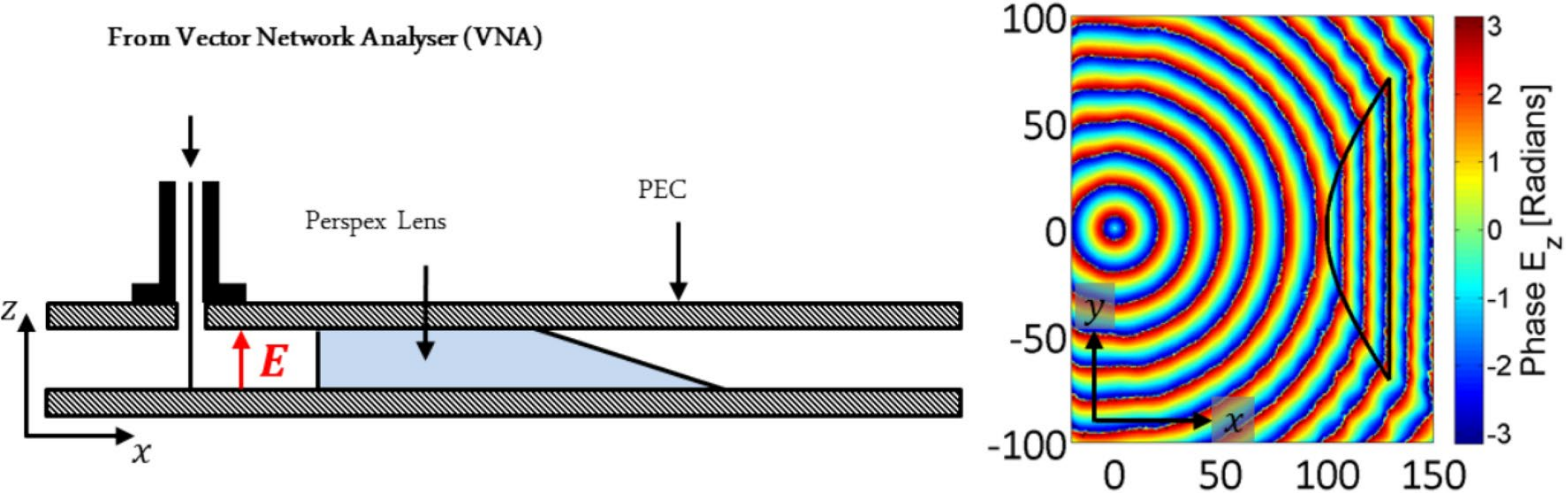
Schematic of the parallel plate waveguide device used for launching planar phase front surface waves onto a metasurface.

In order to determine the reflection coefficient at a straight edge termination at normal incidence a source of surface waves with planar phase fronts is required, since in this case the interference between the forward-propagating incident waves and the backward-propagating reflected waves is analytically trivial. Such a system has been realised using an aspherical Perspex lens to shape the emission from a line antenna source (fed from a port of a VNA) within a parallel plate waveguide into planar phase fronts, Fig. 3. The fundamental transverse electromagnetic (TEM) mode of the parallel plate waveguide is excited from a point within the waveguide by attaching the sheath and pin of a coaxial line to the top and bottom plate of the waveguide respectively. The TEM mode propagates outwards from this point with cylindrical phase fronts in the xy-plane which subsequently pass through a Perspex lens which is uniform in the z-direction and has an aspherical profile described in Eq. (1) in the xy-plane.

1 $$r\left(\theta \right)=\frac{{r}_{0}\left({n}_{2}-{n}_{1}\right)}{{n}_{2}\mathit{cos}\theta -{n}_{1}}$$

Here ${n}_{1}$ is the refractive index of the material filling the waveguide (air in this case), ${n}_{2}$ is the refractive index of the material used to make the lens, $\theta$ is the angle from the x-axis and ${r}_{0}$ is the distance from the point source to the front of the lens at $\theta ={0}^{o}$. This profile modifies the cylindrical phase fronts produces at the source into planar phase fronts at the exit of the lens, which is tapered to avoid reflection within the lens, across a broad frequency range^18^. The end of this waveguide is then placed in contact with the metamaterial surface in order to excite the surface waves via the localised field components produced by diffraction at the waveguide exit.

The near-field of the surface waves supported on the metasurface has been experimentally characterised using a near-field coaxial probe (a terminated length of coaxial cable with the sheath removed from a short length at the end), connected to a second port of the VNA. The probe, which provides a measurement of the E_z_-component of the field, is scanned across the structure by using a three-axis translation stage. The surface wave fields are reconstructed from the magnitude and phase of the complex transmission coefficient between the two ports of the VNA. From this, the reflection coefficient of surface waves incident upon the termination of a metasurface to free space is determined.

**Figure 4:**
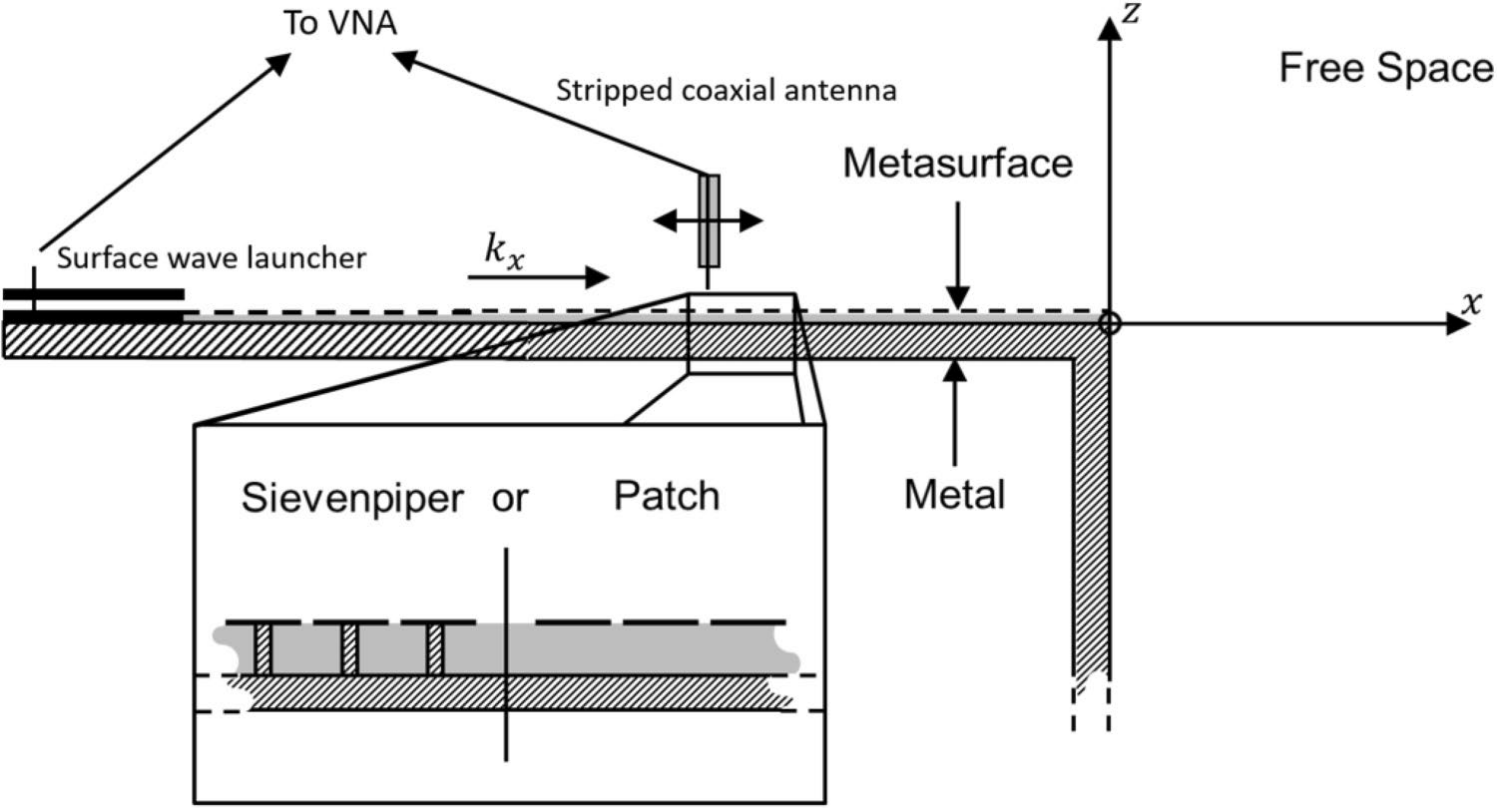
Schematic of the experimental setup including the termination of a metasurface to free space. The shaded region indicates the L-shaped metal support. At the left-hand end of the setup is the surface wave launching device from Fig. 3. The connections between the VNA, the surface wave launcher and the scanning stripped coaxial probe used to map the electric field are also indicated.

The magnitude of the reflection coefficient from a termination depends strongly on the boundary conditions associated with the local environment of the termination^1–3^. The boundary conditions imposed in this case are shown in Fig. 4. A metal boundary extends ‘infinitely’ into the lower half-space connected to the ground plane of the metasurface. Practically this has been achieved by electrically connecting the ground plane of the metasurface to an L-shaped section of aluminium; the electrical connection was created by using a silver loaded epoxy, and then terminating the aluminium with a microwave absorber (Eccosorb VHP-4).

## Experimentally determined reflection coefficient

**Figure 5:**
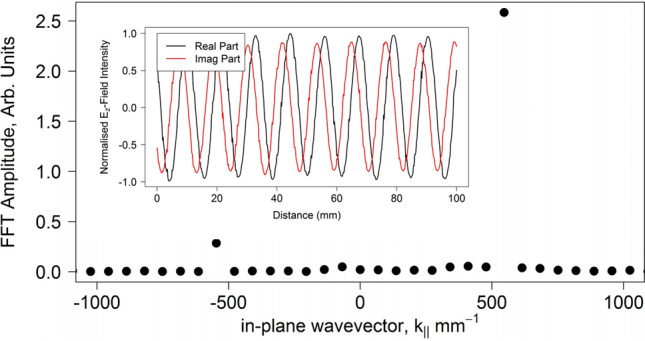
Example of the Fast Fourier Transform (FFT) of the ${E}_{z}$-field showing the relative amplitudes of the forward and backward propagating waves. Inset: Corresponding ${E}_{z}$-field measured using the near-field coaxial probe reconstructed from the time averaged field amplitude and the phase.

The spatial field maps are used to determine the reflection coefficient of surface waves from the discontinuity by utilising the interference between the incident and reflected surface waves. The instantaneous ${E}_{z}$-field is reconstructed by measuring both the time averaged field strength and the phase of the electric field. A fast Fourier transform (FFT) performed on the measured instantaneous ${E}_{z}$-field gives the relative amplitude of the Fourier components present, see Fig. 5. The amplitude reflection coefficient is simply obtained from the ratio of the magnitude of the Fourier component whose wavevector corresponds to the reflected wave equivalent to that of the incident wave. This process is repeated for each frequency across the measurement band of interest.

The spatial length of the field map analysed determines the wavevector resolution within the FFT series, and the spatial resolution determines the maximum wavevector. However, if wavevector components corresponding to the incident and reflected surface waves are not permitted within the FFT series then the incorrect reflection coefficient will be measured. To ensure that the required wavevectors are within the FFT series, a peak-finding algorithm is used to find the position of the interference peaks in the measured field, which has been passed through a smoothing algorithm. The measured field is then truncated at the positions of the first and last peaks to ensure that the optimal spectral length is used.

The reflection coefficient of a surface wave incident upon the termination of a metasurface to free space is related to the confinement of the surface wave in the $z$-direction above the metasurface. This confinement is defined by the imaginary component of the k-vector perpendicular to the interface, ${k}_{\mathrm{z}}$, which is given by ${k}_{\mathrm{z}}^{2}={k}_{0}^{2}-({k}_{\mathrm{x}}^{2}+{k}_{\mathrm{y}}^{2})$ where the total k-vector, ${k}_{0}$ is fixed by the frequency, ω. FEM predictions of the dispersions (ω vs. *k*_x_) of the surface waves supported by each of the two metasurfaces are shown in Fig. 2. As the surface wave dispersion diverges from the light line (i.e., *k*_x_ > *k*_0_), so the decay length of the mode in the z-direction decreases (i.e. *ik*″_*z*_ grows). This increasing confinement of the mode increases the mismatch of the wavevector in the propagation direction, ${k}_{\mathrm{x}}$, of the surface wave by comparison with the k-vector of a wave propagating in free space. However the reflection coefficient of the surface wave is not simply determined by this mismatch of ${k}_{\mathrm{x}}$ but also by the field continuity conditions. There is no electric-field below the metal ground plane at *z* = 0 of the metasurface, in the region *x* < 0, *z* < 0, whilst the electric-field in the free space region has non-zero E-field within the *x* > 0, *z* < 0 region—see Fig. 4. This field distribution plays an important role since even a mode whose in-plane wavevector is equal to that of the light line in the microwave domain (i.e. a surface wave on a flat perfect conductor) when made incident on such a termination has a non-zero reflection coefficient due to this field discontinuity (i.e. a mismatch in wavefunctions at the boundary).

**Figure 6:**
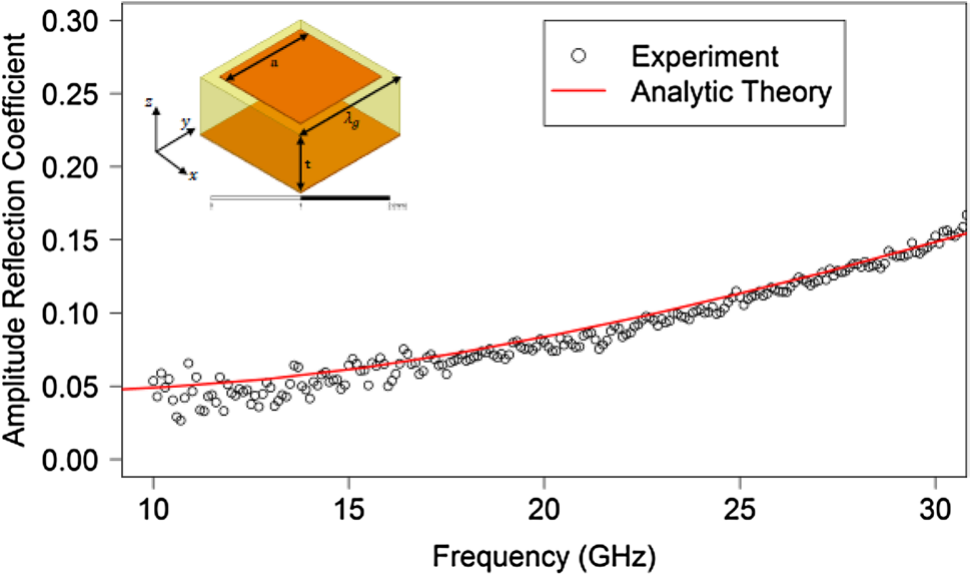
Reflection coefficient on a metallic patch array of the surface wave normally incident on the straight edge termination of the metasurface to free space—experiment (circles) and analytic theory^1^ (line). Inset: Schematic diagram of the unit cell of the square array of square metallic patches.

**Figure 7:**
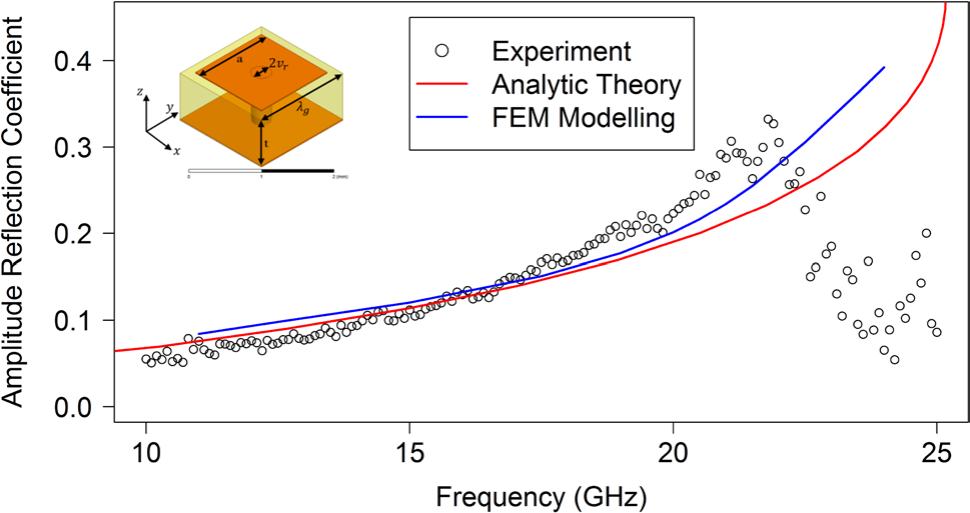
Reflection coefficient of the surface wave on a mushroom-array incident on the termination of the metasurface surface to free space, experiment (circles) and analytic^1^ theory (line). FEM modelling is shown by the blue line. Inset: Schematic diagram of the unit cell of the array.

The amplitude reflection coefficient from experiments of the surface wave incident upon the termination of the metallic patch array is shown in Fig. 6 and for the Sievenpiper ‘mushroom’ array in Fig. 7. In each of these diagrams the reflection coefficient obtained experimentally is shown as open circles whilst the reflection coefficient obtained by using the analytical theory of Chu et al.^1^ is shown by the lines. This analytic theory utilises an auxiliary function which satisfies both the impedance boundary condition and the perfect conductor boundary condition to solve for the electric field around the terminated impedance boundary.

In both cases, at the lower frequencies nearly all of the surface wave is transmitted to free space notwithstanding the very large discontinuity at the edge termination. As the frequency is increased and the surface wave becomes more confined, this transmittance starts to reduce but even at 30 GHz in the patch array case the amplitude reflection coefficient is still around 0.15. Of course, Fig. 2 shows that the wavevector of the surface wave is still within 10% of that of a free photon so this slow decrease of reflectivity with frequency is perhaps to be expected. More strikingly, as revealed by an inspection of the dispersion curve in Fig. 2 for the Sievenpiper case, above about 22 GHz the surface wave dispersion approaches its asymptote and therefore the rate of increase with frequency of the wavevector of the surface wave increases. This leads to an increased mismatch between the wavevector of the surface wave and that of a grazing photon and so the rate of increase in the reflection coefficient correspondingly rises. The agreement between the analytic theory and experimentally determined reflection coefficient for the metallic patch array is good. However the agreement between the analytic theory and experiment for the mushroom-array surface is poor except at low frequencies. FEM models have also been analysed to determine the reflection of the surface waves by modelling the whole metasurface structure in a single strip with appropriate boundary conditions (perfect magnetic) to simulate an infinitely wide structure. In this model the surface wave is launched using a parallel plate waveguide to simulate the experimental plane wave launcher and the field analysed in the same way as for the experiment. The FEM modelling, shown by the blue line in Fig. 7, agrees with the analytic theory at low frequencies but also diverges at higher frequencies while remaining in reasonable agreement with the experiment. The reason for the disagreement is that the impedance boundary condition does not take into account the power flowing below the interface of the metamaterial. This power flow is much greater for the Sievenpiper mushroom structure than for the patch array studied previously and therefore the impedance boundary simplification is less suitable.

Further note that there is a significant increase in the non-radiative loss of the surface mode on the mushroom array as the dispersion curve diverges from the light line (Fig. 2) which prevents the accurate determination of the reflection coefficient at high wavevectors using the current method. The analysis used in our calculation of the reflection amplitude assumes that the loss associated with the propagation of the surface wave is negligible, i.e. Im $({k}_{x})\approx 0$. Once the in-plane wavevector, $k_{x} = k^{\prime\prime}_{x} + ik^{\prime\prime}_{x}$, has a significant imaginary part then determining the reflection coefficient from the FFT is no longer trivial. This is due to the broadening of the peaks in the FFT spectrum such that their relative ratios are no longer equal to the amplitude reflection coefficient. In addition, due to losses, the strong interference that yields the reflection coefficient is found only very near the end termination. This effect gives rise to the apparent decrease in the measured reflection coefficient above 22 GHz in Fig. 7, which is not in agreement with either the analytic theory or the FEM modelling.

## Conclusions

The normal incidence surface wave reflection coefficient at the termination of a metasurface to free space for both an array of metallic patches on a dielectric coated ground plane and a mushroom array has been determined. For the patch-array metasurface both an analytical model and finite element predictions agree well with the experimental data. However for the mushroom metasurface the analytical model and the FEM model diverge from the experimental data at higher frequencies. This divergence is caused by the surface wave properties of the mushroom metasurface, most notably the decrease in the surface wave propagation length at frequencies close to the asymptote of the fundamental TM mode. The current method for measuring the surface wave reflection assumes that the propagation length of the surface wave is much larger than the sample length. The reflection coefficient of amplitude is rather small, about 5% at 10 GHz for both structures, and increases with the progressive confinement of the surface mode at higher frequencies. This increase in the reflection coefficient is caused by both the momentum mismatch of the surface wave compared with the freely propagating modes and the different field distributions of the two modes.
